# Controlled Drug Release Using Chitosan-Alginate-Gentamicin Multi-Component Beads

**DOI:** 10.3390/ma15217682

**Published:** 2022-11-01

**Authors:** Kyung Hee Park, Yeon Woo Choi, Heejoo Ryu, Hyoung Jae Lee, Jae-Hak Moon, Ho-Jun Song, Yeong-Joon Park

**Affiliations:** 1 Department of Dental Materials and Hard-Tissue Biointerface Research Center, School of Dentistry, Chonnam National University, Gwangju 61186, Korea; 2 School of Materials Science & Engineering, Chonnam National University, Gwangju 61186, Korea; 3 Department of Food Science and Technology, Chonnam National University, Gwangju 61186, Korea

**Keywords:** alginate, chitosan, controlled release, bioactivity, gentamicin

## Abstract

This study aimed to develop improved multi-component beads with controlled, sustained delivery of antibiotics, such as gentamicin (GM). Antibiotic-loaded beads with rapid-release and the sustained-release system can be used for bone restoration. Single and multi-component beads were prepared by gelation using various combinations of chitosan and calcium chloride as cationic components and alginate and citric acid as anions. GM release was also controlled by crosslinking using citric acid. The optimum beads were obtained using 5% or 2% sodium alginate, 3% chitosan, and 0.1 mol/L citric acid. The beads were characterized by FTIR, TG-DTG, swelling behavior, and SEM. All GM-loaded beads revealed good antimicrobial activity. The rate and kinetics of release in the phosphate buffer solution were controlled by changing the amount of chitosan in the calcium chloride solution and using citric acid as the crosslinking agent. Crosslinked beads were prepared for the release of about 80% of the loaded drug within 24 h. The study concluded that the chitosan-alginate beads provided faster GM release but crosslinking with citric acid was efficient for sustained-release beads containing gentamicin.

## 1. Introduction

Drug delivery systems frequently use biodegradable, biocompatible, and natural biopolymers to control the kinetics of drug release [1]. Chitosan and alginate are the most representative materials used in beads and encapsulation for drug release because they are non-toxic, biodegradable, and biocompatible [2,3,4,5]. However, their antibacterial characteristics are unsatisfactory for preventing infections after bone surgery. Therefore, additional material is necessary for the targeted delivery of drugs. Polymers have been studied as drug carriers to create sustained and controlled antibiotic-release systems for preventing infections [6,7]. Hydrogels based on natural or synthetic polymers, including antibiotics such as ciprofloxacin [8], gentamicin [9,10], vancomycin [11], and ampicillin [12], have been extensively studied. Several reports demonstrate that gentamicin (GM) can be an efficient antibiotic for preventing various bacterial infections because it acts against a broad spectrum (gram-positive and gram-negative) of bacteria [13,14]. GM is an aminoglycoside antibiotic consisting of five components (C_1_, C_1a_, C_2_, C_2a_, and C_2b_) [15]. Quantitative analysis of gentamicin using liquid chromatography-tandem mass spectrometry (LC-MS/MS) has been investigated. Total gentamicin is the sum of the concentrations of five components: C_1_, C_1a_, C_2_, C_2a_, and C_2b_. 

Recently, polyelectrolyte complexes have been used for designing drug delivery systems [16,17,18,19]. Chitosan can be used to prepare gels with anionic counter ions of sodium alginate by crosslinking. Alginate plays a key role in developing biomaterials for controlled drug release [20]. Hydrogels have the advantages of sustained and controllable release, the capability of localized use (thus avoiding the side effects of systemic delivery), and biocompatibility contrary to traditional pharmaceutical formulations. Moreover, synergic combinations of ingredients can be used against resistant bacteria [21]. 

Sionkowska et al. reported that gentamicin release from complexes of chitosan and collagen could be controlled and localized by variations in the chitosan content [22,23]. Drug release from polymeric matrix occurs by three mechanisms: (1) polymer erosion, (2) diffusion through swollen materials, and (3) diffusion from the surface of materials [24]. 

Daly and Knorr reported that macromolecular chitosan rapidly binds onto the surface of alginate droplets, but there was a limitation of diffusion from the inner core. Chitosan solution and CaCl_2_ were used for alginate gelation [25] to increase the stability of the alginate-chitosan complex. There are few reports on improving drug loading and release behaviors from alginate and alginate-chitosan beads.

In this study, we prepared alginate-chitosan-gentamicin beads and alginate-gentamicin beads crosslinked with citric acid (CA) to develop a stable, non-toxic polymer complex to control drug release properties. CA can form crosslinked beads by ionic interaction with positively charged Ca-alginate. Gentamicin, a broad-spectrum antibiotic used in this study, has effective antibacterial activity against many strains of Gram-negative (e.g., *E. coli*) and some strains of Gram-positive (e.g., *S. mutans*) bacteria; moreover, it shows cost-efficacy and stability in thermal properties. The surface morphology of the beads was characterized by scanning electron microscopy (SEM), and loss of weight, swelling behavior, and the release profile using UV/Vis spectrophotometer and LC-tandem mass spectrometer (LC/MS/MS) were determined.

## 2. Materials and Methods

### 2.1. Materials

Sodium alginate (M.W. 200 kDa), calcium chloride dihydrate, gentamicin sulfate (GM), citric acid (CA), and chitosan (75–85% degree of deacetylation, M.W. 50–190 kDa) were obtained from Sigma-Aldrich (St. Louis, MO, USA). Phosphate buffer saline (PBS) was purchased from Gibco (Carlsbad, CA, USA). Acetonitrile and trifluoroacetic acid were purchased from Biosolve (Valkenvaard, The Netherlands), and formic acid was obtained from Merck (Darmstadt, Germany).

### 2.2. Preparation of Chitosan-Alginate Beads 

**Scheme 1:**
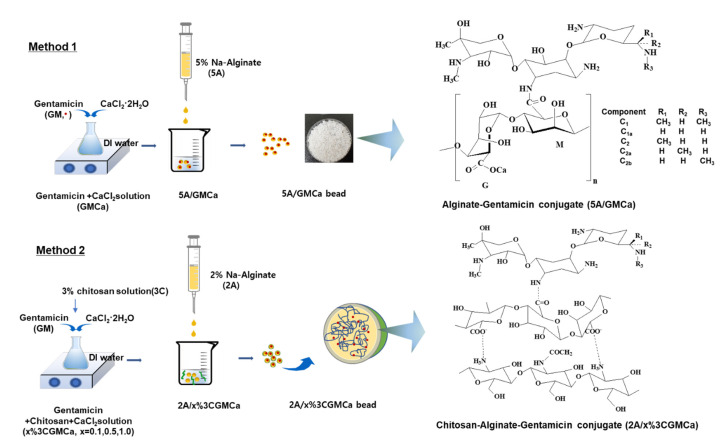
Schematic illustration of reaction mechanism involved in the formation of alginate-gentamicin conjugate (5A/GMCa) and alginate-chitosan-gentamicin conjugate (2A/x%3CGMCa) beads.

The beads were prepared by two methods, as shown in Scheme 1, and named depending on their multi-components: chitosan, calcium chloride dihydrate, alginate, and citric acid. Chitosan-alginate beads were obtained by gelation. A homogenous sodium alginate solution in distilled water (2 and 5 %, *w*/*v*) was used as dope. Chitosan (3%, *w*/*v*) was dissolved in 2 % (*v*/*v*) acetic acid at 60 °C and named 3C. Homogenous aqueous solutions of 3C and calcium chloride dihydrate (0.1 N CaCl_2_·2H_2_O, named Ca) with different chitosan solution content were used as coagulation solutions. The mixing of the solution was performed for 2 h. Alginate solution (50 mL) was added dropwise through a 21 G needle into the coagulation solution (200 mL) while stirring at 150 rpm. The obtained spherical beads remained for 6 h in the coagulation solution with stirring. Then, beads were filtered, washed ten times with distilled water, and freeze-dried for 24 h. 

### 2.3. Preparation of Alginate-Gentamicin and Alginate-Chitosan-Gentamicin Beads

Alginate-gentamicin (5A/GMCa) conjugates and alginate-chitosan-gentamicin (2A/x%3CGMCa) beads were prepared by gelation (Scheme 1). A 50 mL of 5% aqueous sodium alginate solution was dropped into 200 mL of 0.1 N CaCl_2_·2H_2_O solution (named 5A). For preparing the 5A/GMCa beads, 50 mL of 5% sodium alginate solution was dropped into 200 mL of 0.1 N CaCl_2_·2H_2_O solution mixed with 0.05 g of gentamicin powder, and 5A/GMCa/CA was prepared from 5A/GMCa beads by 5 h post-crosslinking treatment in citric acid. The prepared beads were washed 10 times with distilled water. 

Alginate-chitosan beads were produced by dropping 2% sodium alginate into 3% chitosan solutions of different chitosan contents in 200 mL of 0.1 N CaCl_2_·2H_2_O solution to make 2A/0.1%3C (including 0.1 wt.% of 3% chitosan solution), 2A/0.5%3C (including 0.5 wt.% of 3% chitosan solution), and 2A/1.0%3C (including 1.0 wt.% of 3% chitosan solution). Likewise, alginate-chitosan beads loaded with gentamicin (0.05 g) were named 2A/0.1%3CGMCa, 2A/0.5%3CGMCa, and 2A/1.0%3CGMCa. The alginate-chitosan beads were rinsed with distilled water, filtrated, and then freeze-dried for 24 h.

### 2.4. Characterization of the Beads

The surface morphologies of the beads were studied using a field-emission scanning electron microscope (FE-SEM; S-4700, Hitachi, Tokyo, Japan). The beads size distribution was calculated using an optical analyzer (Camscope, Sometech Co. Ltd., Seoul, Korea). The FTIR spectra for the beads were obtained using an FTIR spectrometer equipped with an attenuated total reflection module (Tensor I, Bruker, Billerica, MA, USA) in a 500–4000 cm^−1^ range with a resolution of 1 cm^−1^. The thermal properties of the sodium alginate, gentamicin sulfate, alginate-chitosan-gentamicin (2A/x%3CGMCa), and alginate-gentamicin (5A/GMCa) beads were examined by thermogravimetric analysis (TGA; Mettler Toledo, Greifensee, Switzerland) in the range from 30 °C to 800 °C with a heating rate of 5 °C/min in nitrogen atmosphere (flow rate of 40 mL/min).

### 2.5. Swelling Test

First, 10 mg of beads were placed in 6 wells containing 2 mL of PBS solution at 37 °C with 150 rpm shaking. For measuring the swelling ratio, the beads were immersed in PBS solution. The wet weight of the swollen beads was measured at periodic intervals immediately after removing excess water with filter paper. The swelling ratio was calculated by the following Formula (1) [26]:

(1) $$\mathrm{SR}\;\left(\%\right)=\frac{W_{x}-W_{i}}{W_{i}}\times 100$$

Here, SR, *W_x_*, and *W_i_* denote the swelling ratio, the weight of wet beads at the determined time, and the initial weight of beads, respectively.

For evaluating PBS uptake, the beads were immersed in PBS solution for 24 h, and the wet beads were weighed. The PBS uptake was calculated by the following Formula (2), in which *W*_1_ and *W_i_* denote the weight of wet beads and the initial weight of dry beads, respectively.

(2) $$\mathrm{PBS}\;\mathrm{uptake}\;\left(\%\right)=\left(\frac{W_{1}-W_{i}}{W_{i}}\right)\times 100$$

The PBS content of beads after being soaked in PBS for 24 h was calculated by the following Formula (3):

(3) $$PBS\;content=\frac{W_{1}-W_{i}}{W_{1}}$$

The experiments were performed in triplicate.

### 2.6. Antimicrobial Assay

The antibacterial activity of gentamicin-loaded beads was evaluated following the viable-cell count method [27]. The tested bacteria were *Escherichia coli* (*E. coli*) obtained from Korean Collection for Type cultures (KCTC, 1682) and *Staphylococcus mutans* (*S. mutans*) from the American Type Culture Collection (ATCC, 25175). Briefly, 17 mg of beads were transferred into a transwell, and the beads were immersed in each well of 24 plates containing 1 mL of *E. coli*-inoculated PBS (1 × 10^5^/mL). This plate was incubated for 24 h at 37 °C. Then, aliquots of 100 uL were collected from each supernatant, plated on nutrient agar plates, and incubated for 24 h at 37 °C. After 24 h, colony formation was examined to determine antibacterial activity. Meanwhile, 5% alginate (5A) beads and alginate-chitosan beads without gentamicin-loading (2A/x%3C) were used as the control. Antibacterial activity was also performed for *S. mutans* plated on BHI agar plates using the same procedure. All experiments were performed in triplicate.

### 2.7. Release Behavior of Gentamicin from Beads

To study the in vitro release of GM from alginate beads and alginate-chitosan beads, the beads were soaked in PBS and incubated at 37 °C. At various time points, 1 mL of extract was taken to measure the amount of released GM. After each measurement point, the wells were refilled with fresh PBS solution. To measure the spectrophotometric value after color reaction with an *o*-phthal aldehyde solution, 1% (*w*/*v*) *o*-phthal aldehyde solution was prepared by dissolving in 5 mL of methanol. Then, 20 μL of 1% *o*-phthal aldehyde solution was mixed in 2 mL of released GM solution. The released GM solution was examined using a UV/Vis spectrophotometer at a wavelength of 230 nm [28]. 

The total amount of released gentamicin was evaluated with an LC/MS/MS. An ACQTTY ultra-performance liquid chromatography (UPLC) H-Class system (Waters, Manchester, UK), coupled to a quadrupole time-of-flight tandem mass spectrometer (XEVO-G2XSQTOF system, Waters, Manchester, UK), was used. The separation was performed on a UPLC with an ACQTTY UPLC^®^ HSS T3 column (100 mm × 2.1 mm, 1.8 μm, Waters, Milford, MA, USA), a column temperature of 40 °C, a flow rate of 0.35 mL/min, an autosampler temperature of 15 °C, and an injection volume of 1 μL. The mobile phase was composed of 0.1% (*v*/*v*) aqueous trifluoroacetic acid (up to pH 2.5 with ammonia water, solvent A) and 100% acetonitrile with 0.1% formic acid (solvent B), using the following gradient elution program: 100% solvent A maintained for 0 to 6 min; solvent B increased linearly to 100% for 6 to 10 min; 100% solvent B held for 10 to 14 min; solvent A immediately returned to 100% and re-equilibrated for 15 to 20 min. Tandem MS (MS/MS) data were obtained from ionization in the electrospray ionization (ESI) positive mode, a capillary voltage of 2.5 kV, source temperature of 130 °C, desolvation temperature of 250 °C, desolvation gas flow of 600 Lh^−1^, cone gas flow rate of 50 Lh^−1^, cone voltage of 40 V, and multiple reaction monitoring (MRM) transitions as *m/z* 478.3 → 322.2/157.3 for Gentamicin C_1_, *m/z* 450.3 → 322.2/160.3 for Gentamicin C_1a_, and *m/z* 464.3 → 322.2/160.3 for Gentamicin C_2_, C_2a_, and C_2b_.

## 3. Results and Discussion

**Figure 1:**
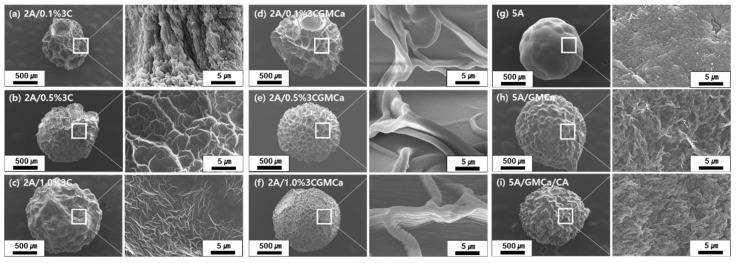
SEM images of alginate-chitosan (2A/x%3C; x = 0.1, 0.5, 1.0) beads with different chitosan content (**a**–**c**) and gentamicin-loaded alginate-chitosan (2A/x%3CGMCa; x = 0.1, 0.5, 1.0) beads with different chitosan content (**d**–**f**). Alginate beads: (**g**) alginate-only (5A), (**h**) with gentamicin (5A/GMCa), (**i**) with gentamicin, and then crosslinked with citric acid (5A/GMCa/CA). The right-side images are magnified views of each left-side image.

Figure 1 shows the surface morphology of the gentamicin-loaded alginate and alginate-chitosan beads with different chitosan content. The beads present a spherical shape, and the right-side images are high-magnification images of the square part of each sample. Their surface consists of grooves and ridges that create a rough surface. In Figure 1a, the surface of 2A/0.1%3CGMCa beads is more granular and grooved, and the surface of 2A/1.0%3CGMCa (Figure 1c) is smoother than that of 0.1% (Figure 1a) and 0.5% (Figure 1b). This is induced by the swollen bulky structure produced by increased chitosan concentration. Figure 1d–f shows gentamicin-loaded alginate-chitosan beads with different chitosan content. An increase in chitosan content produced small holes on the bead surface. In Figure 1g, the 5A beads show a smooth surface. The gentamicin-loaded 5A/GMCa (Figure 1h) shows a rough surface like pop-con. Evident differences are observed between gentamicin-unloaded and -loaded alginate beads. The 5A/GMCa/CA beads crosslinked with CA show rougher surfaces than 5A/GMCa. These results can predict the effect on surface roughness, the shape of beads, and gentamicin release from beads. 

**Figure 2:**
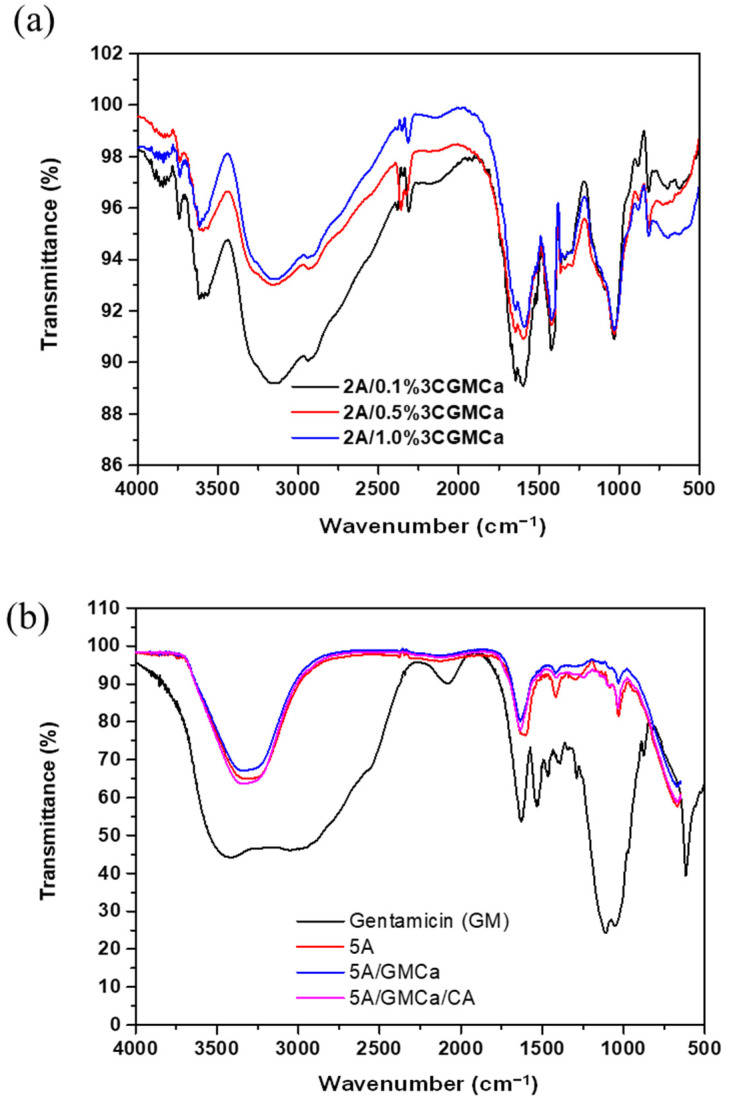
FTIR spectra of (**a**) gentamicin-loaded alginate-chitosan (2A/x%3CGMCa; x = 0.1, 0.5, 1.0) beads with different chitosan content. (**b**) Alginate beads: alginate-only (5A), with gentamicin (5A/GMCa), and with gentamicin and then crosslinked with citric acid (5A/GMCa/CA).

Figure 2 describes FT-IR spectra of 2A/x%3CGMCa beads (a) and 5A/GMCa and 5A/GMCa/CA beads (b). FT-IR spectra shown in Figure 2a confirm the presence of hydroxyl group and N–H stretching vibration at 3339 cm^−1^ overlapped by N–H stretching of chitosan. The C–H stretching peaks of sodium alginate and chitosan at 2935 cm^−1^ are shown. The absorption peak of C=O of sodium alginate and amide I band of chitosan appeared at 1630 cm^−1^. The amide II band, due to N–H bending, appeared at 1640 cm^−1^, which is overlapped by the amide I band. The carboxyl group of sodium alginate shows another C–O absorption at 1430 cm^−1^. The N–H bending vibration peak was observed at 832 cm^−1^. FT-IR spectra of alginate-chitosan beads confirmed a bipolymeric formation. 

In the FTIR spectra of gentamicin sulfate (Figure 2b), the typical absorption bands at 1630, 1529, and 1235 cm^−1^ could be assigned as the amide I, amide II, and amide III bonds of gentamicin, respectively. The peak observed at 1108 cm^−1^ was due to the HSO_4_^−1^ group. The peak observed at 617 cm^−1^ was due to the SO_2_ band. The peak of gentamicin at 1541 cm^−1^ was present with low intensity, suggesting successful loading of gentamicin into alginate beads.

**Figure 3:**
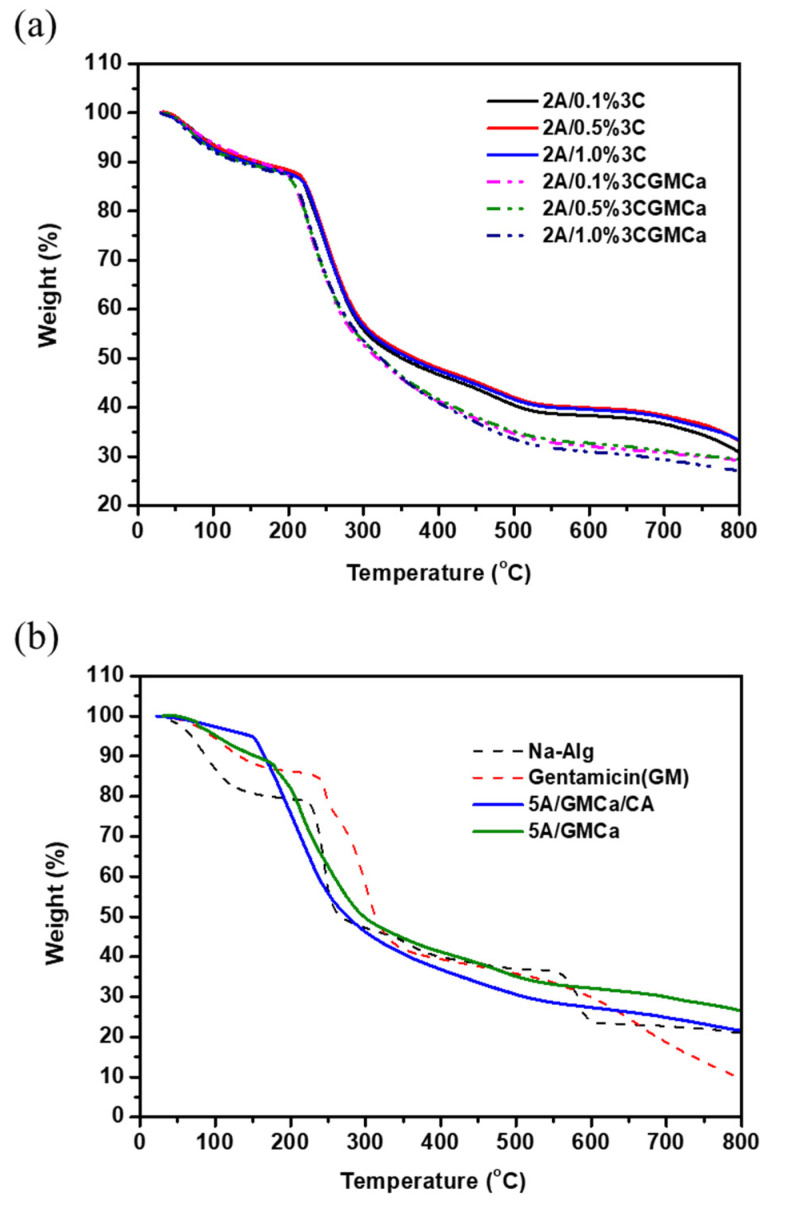
TGA thermograms of (**a**) alginate-chitosan (2A/x%3C; x = 0.1, 0.5, 1.0) beads with different chitosan content, and (**b**) alginate beads: alginate-only (5A), with gentamicin (5A/GMCa), and with gentamicin and then crosslinked with citric acid (5A/GMCa/CA).

The TGA curves are shown in Figure 3. As shown in Figure 3a, the thermograms of 2A/x%3C beads were very similar, indicating that the thermal stability of the beads was not affected meaningfully by the difference in chitosan content. For 2A/x%3C beads, the total weight loss was 12~13% and 37~38% at 205 °C and 260 °C, respectively, and a weight loss of 12~13% was detected at 480 °C. For gentamicin-loaded beads (2A/x%3CGMCa), the thermal stability was also similar to the changes in chitosan content. For the 2A/x%3CGMCa beads, the weight loss was 50~52% and 6~7% at 250 °C and 470 °C, respectively, and 12~13% weight loss occurred at 480 °C. Figure 3b shows the thermograms of sodium alginate, gentamicin sulfate, 5A/GMCa, and 5A/GMCa/CA beads. For the 5A/GMCa and 5A/GMCa/CA beads, the weight loss was 45~52% and 20~30% at 220 °C and 420 °C, respectively.

**Figure 4:**
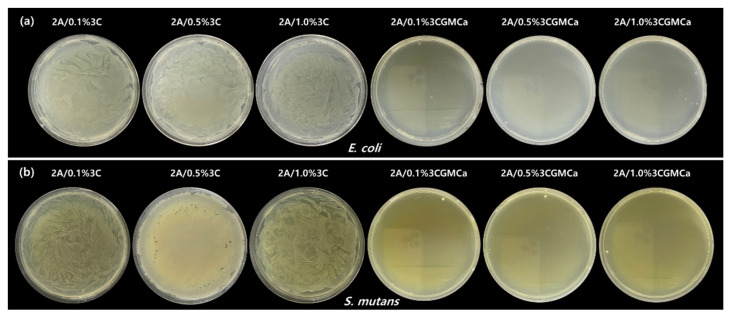
Assays were conducted against gram-negative *E. coli* (**a**) and gram-positive *S. mutans* (**b**). An antibacterial effect was observed in the agar plates inoculated with 100 μL of supernatant PBS solution immersed with chitosan-alginate-gentamicin (2A/x%3CGMCa, x = 0.1, 0.5, 1.0) beads containing different content of chitosan. There was no antibacterial activity by the extract from chitosan-alginate beads (2A/x%3C, x = 0.1, 0.5, 1.0).

Figure 4 shows the antimicrobial activity of GM-unloaded beads (2A/x%3C) and GM-loaded beads (2A/x%3CGMCa) against *S. mutans* and *E. coli*. Chitosan has been widely used as antibacterial material owing to its positively charged moiety. However, the chitosan-alginate beads exhibited no bacteria inhibition capacity, as illustrated in Figure 4. We conjectured that the positive amine group of chitosan reacted with the negative carboxylic group of alginates, by which the positively charged groups of chitosan reduced their availability to interact with bacterial cell walls [29]. The results demonstrate that bacteria could not colonize meaningfully after contact with the GM extract eluted from 2A/x%3CGMCa beads, which was associated with the bactericidal activity of the alginate-chitosan-GM beads. All gentamicin-loaded alginate-chitosan beads showed antibacterial effects because of corroborating with the gentamicin bound to the alginate and alginate-chitosan chains. 

**Figure 5:**
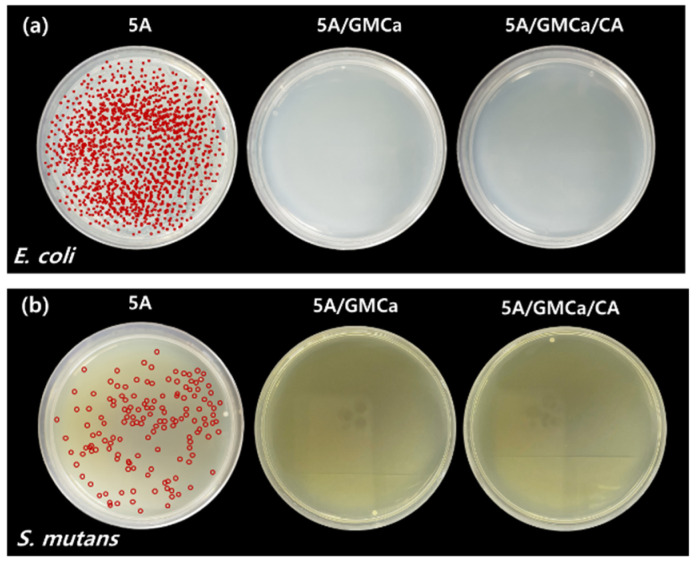
Optical photos of antibacterials effect of 5A, 5A/GMCa, and 5A/GMCa/CA (**a**) against *E. coli* and (**b**) against *S. mutans*. An antibacterial effect was observed in the agar plates inoculated with 100 uL PBS solution containing gentamicin-loaded alginate (5A/GMCa) beads and gentamicin-loaded alginate crosslinked with citric acid (5A/GMCa/CA) beads while there was no antibacterial activity by the extract from alginate-only beads (5A) as demonstrated by the appearance of numerous colony formations on the agar plates (marked with red circles).

Figure 5 compares the efficiency of 5A (control), 5A/GMCa, and 5A/GMCa/CA beads against gram-positive *S. mutans* and gram-negative *E. coli*. The neat alginate (5A, control) did not exhibit any antibacterial killing ability against both tested bacteria, *S. mutans* and *E. coli*. All gentamicin-loaded beads (5A/GMCa, 5A/GMCa/CA) showed suitable antibacterial activity against *E. coli* and *S. mutans*. 

**Figure 6:**
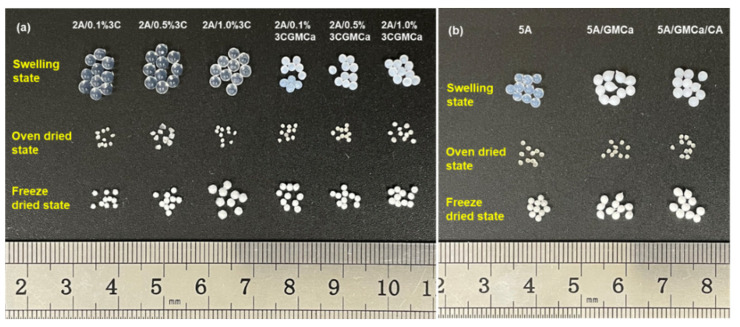
Photographs of the beads in different states: (top) swelling state, (middle) oven-dried state, and (bottom) freeze-dried state; (**a**) alginate-chitosan (2A/x%3C, x = 0.1, 0.5, 1.0) and GM-loaded alginate-chitosan (2A/x%3CGMCa, x = 0.1, 0.5, 1.0) beads, and (**b**) alginate (5A), GM-loaded alginate (5A/GMCa), and GM-loaded alginate crosslinked with citric acid (5A/GMCa/CA) beads.

Figure 6a shows the optical images of GM-unloaded alginate-chitosan beads (2A/x%3C, x = 0.1, 0.5, 1.0) and GM-loaded beads (2A/x%3CGMCa), classified by swelling condition (swelled, oven-dried, and freeze-dried). In the swelled condition, 2A/x%3C beads were semi-transparent with a spherical shape. Oven-dried beads became opaque, and the size reduced noticeably with a less spherical shape. However, when the beads were freeze-dried, they shrank less than the oven-dried beads. Figure 6b shows optical images of alginate beads (5A, 5A/GMCa, 5A/GMCa/CA) in swelled conditions. Notably, adding GM in the alginate beads (5A/GMCa) and CA crosslinking of the alginate beads (5A/GMCa/CA) did not induce bead size reduction. 

**Figure 7:**
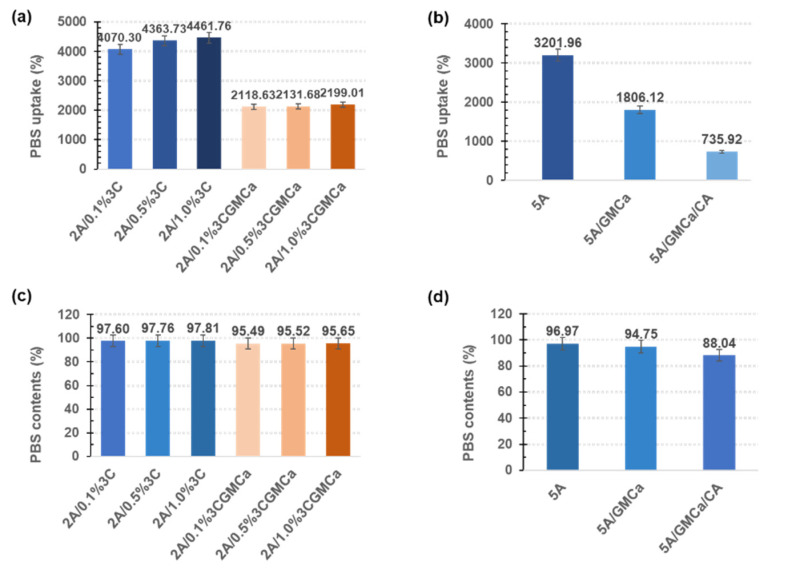
Uptake of PBS for (**a**) alginate-chitosan (2A/x%3C; x = 0.1, 0.5, 1.0) beads and GM-loaded alginate-chitosan (2A/x%3CGMCa; x = 0.1, 0.5, 1.0) beads with different chitosan content; for (**b**) alginate (5A), GM-loaded alginate (5A/GMCa), and GM-loaded alginate with citric acid crosslinked (5A/GMCa/CA) beads after equilibration for 24 h. Content of PBS for (**c**) alginate-chitosan (2A/x%3C, x = 0.1, 0.5, 1.0) and GM-loaded alginate-chitosan (2A/x%3CGMCa, x = 0.1, 0.5, 1.0) beads; for (**d**) alginate-only (5A), GM-loaded alginate (5A/GMCa), and GM-loaded alginate crosslinked with citric acid (5A/GMCa/CA) beads after equilibration for 24 h. Measurements were performed in triplicate.

Figure 7a shows the PBS uptake of the beads for 24 h. The average amount of PBS uptake in alginate-chitosan (2A/x%3C, x = 0.1, 0.5, 1.0) beads ranged from 4070.30 to 4461.76%. The GM-loaded beads (2A/x%3CGMCa) showed 2118.63 to 2199.01% of PBS uptake ability. The degree of PBS uptake in GM-loaded beads (2A/x%3CGMCa) was about half compared to that of GM-unloaded beads. Figure 7b shows the PBS uptake of alginate beads (5A, 5A/GMCa, 5A/GMCa/CA). The PBS uptake of 5A was 3201.96%, that of GM-loaded beads (5A/GMCa) was 1806.12%, and even less amount of PBS uptake was observed at 5A/GMCa/CA as 735.92%. The PBS content is shown in Figure 7c,d. The PBS content of the beads in the swelled state ranged from 97 to 95% of the weight of freeze-dried alginate-chitosan beads. This high PBS absorption property is advantageous for GM release from GM-loaded beads (2A/x%3CGMCa). The PBS contents of alginate beads were in the order of 5A (96%) > 5A/GMCa (94%) > 5A/GMCa/CA (88%) (Figure 7d). These results are attributed to the difference in the surface structure of the beads and the interaction between GM structure with hydrophilic -OH, NH_2_, and -COOH groups of alginate or alginate-chitosan beads.

**Figure 8:**
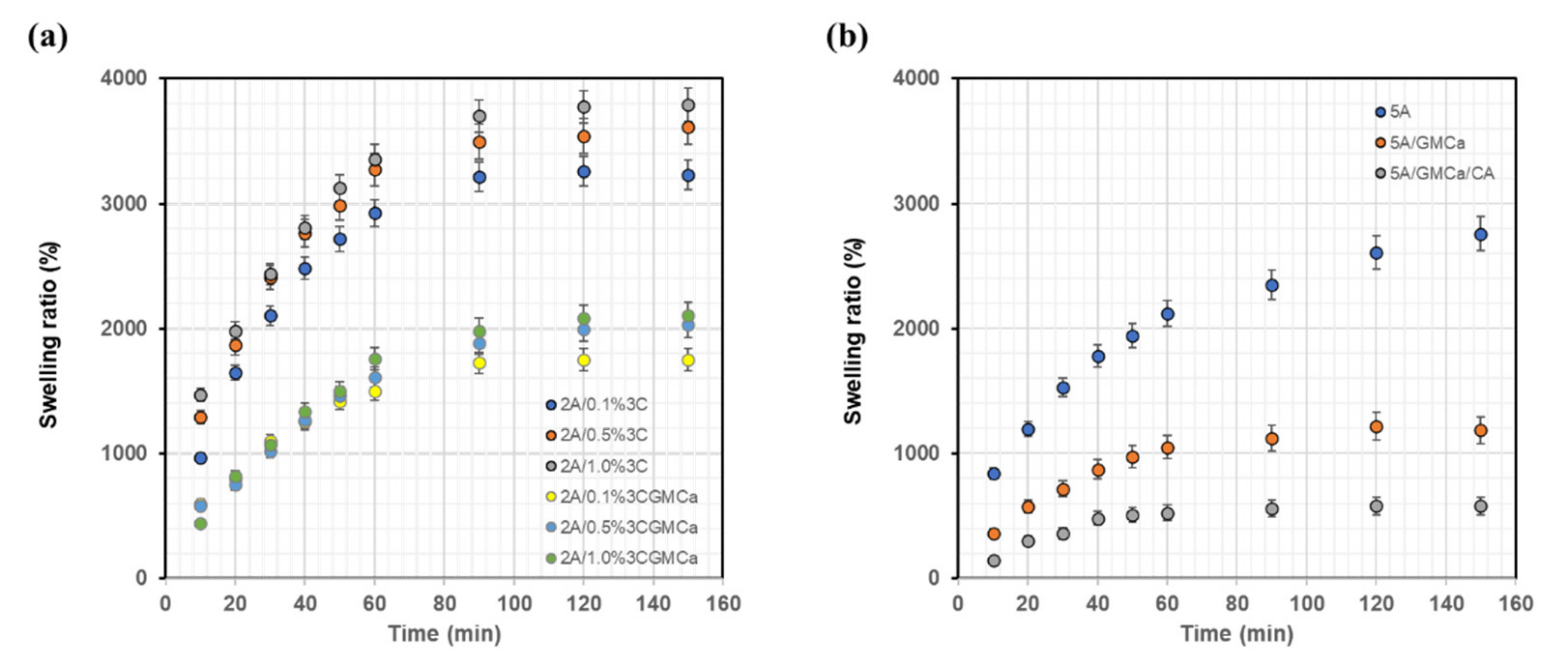
The swelling ratio of the beads as a function of immersion time in PBS solution: (**a**) alginate-chitosan (2A/x%3C, x = 0.1, 0.5, 1.0) and GM-loaded alginate-chitosan (2A/x%3CGMCa, x = 0.1, 0.5, 1.0) beads, and (**b**) alginate (5A), GM-loaded alginate (5A/GMCa), and GM-loaded alginate crosslinked with citric acid (5A/GMCa/CA) beads.

The swelling ratios (SR) of the 2A/x%3C and 2A/x%3CGMCa beads in 150 min are presented in Figure 8a. The beads absorbed PBS 9 to 14 times of weight compared to the freeze-dried beads (SR ≈ 970–1468 %) in the first 10 min. The SR increased steadily until 90 min, and the PBS absorbed bead weights after 150 min were 32 to 38 times the weight of freeze-dried beads (SR ≈ 3228–3793%). This result was similar to previous reports that the SR of the gelatin/chitosan samples ranged from 300 to 6000 % after 150 min [26]. Noteworthy that the SR became higher as the chitosan content in the beads increased from 0.1% to 1.0%. Figure 8b shows the SR of 5A, 5A/GMCa, and 5A/GMCa/CA beads. The SR after 150 min was in the decreasing order of 5A (2760%), 5A/GMCa (1186%), and 5A/GMCa/CA (579%). These findings indicate that the highest PBS content of 5A is due to the existence of more NH_2_ and -OH groups compared to GM-loaded alginate beads and crosslinked 5A/GMCa beads. Especially, 5A/GMCa/CA showed the lowest SR due to a strong chemical network formed after crosslinking.

**Figure 9:**
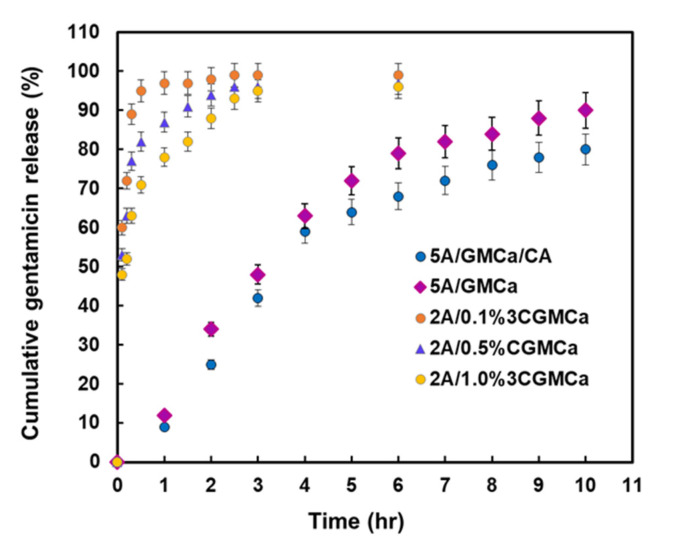
The cumulative release of gentamicin from the beads as a function of immersion time in PBS solution: (top to down) GM-loaded alginate beads crosslinked with citric acid (5A/GMCa/CA), GM-loaded alginate beads (5A/GMCa), and GM-loaded alginate-chitosan beads (2A/x%3CGMCa, x = 0.1, 0.5, 1.0).

GM release from the swollen beads was induced by the interaction between PBS and gentamicin in the polymer in Figure 9. GM released fast initially and reached a plateau after 3 h and 9 h for alginate-chitosan beads (2A/x%3CGMCa) and alginate beads (5A/GMCa, 5A/GMCa/CA). The cumulative GM release was faster from alginate-chitosan-GM (2A/x%3CGMCa, x = 0.1, 0.5, 1.0) beads than alginate-GM beads (5A/GMCa, 5A/GMCa/CA). The GM release rate decreased significantly after 3 h for 2A/0.1%3CGMCa compared to 2A/0.5%3CGMCa and 2A/1.0%3CGMCa. This fast GM release resulted because the electrostatic interactions between the cationic chitosan and GM do not work. In the 5A/GMCa and 5A/GMCa/CA beads, alginate has COO- groups and GM has NH_2_ and -OH groups in the structure. The electrostatic interactions between the alginate and GM delay the release of GM from alginate-GM beads. The release rate of GM was slow, and 90% of GM was released after 9 h. The release of GM from 5A/GMCa beads was higher than that from the 5A/GMCa/CA because of the strong crosslinking function of citric acid between alginate and GM.

**Figure 10:**
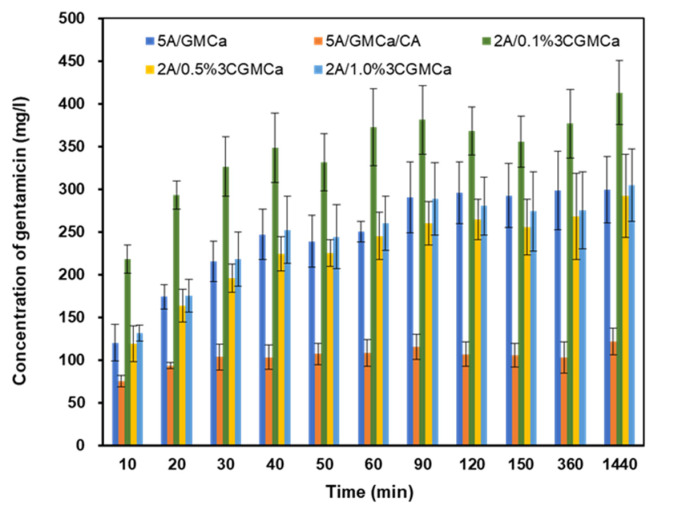
The total amount of gentamicin released from GM-loaded alginate-chitosan (2A/x%3CGMCa, x = 0.1, 0.5, 1.0) beads and GM-loaded alginate beads without/with citric acid (5A/GMCa, 5A/GMCa/CA) as a function of immersion time in PBS solution. Error bars represent the standard deviation from three samples per sample group.

Liquid chromatography-mass spectrometry (LC-MS) was very efficient for identifying and quantifying GM release from the beads. Using the standard concentrations of 3.125, 6.25, 12.5, 25, 40, 100, 250, and 500 mg/L, the concentration of GM in extract solution after 0 to 24 h was quantified. As shown in Figure 10, the GM release increased sharply until 90 min. Then, a slow and gradual release was observed. The 5A/GMCa/CA beads showed the lowest concentration of GM release. The 2A/0.5%3CGMCa, 2A/1.0%3CGMCa, and 5A/GMCa beads showed similar release patterns (*p* > 0.05). The 2A/0.1%3CGMCa beads had the highest GM release profile. These results demonstrate that the GM release is attributed to the difference in surface roughness and chemical structure of chitosan, alginate, and GM conjugates.

## 4. Conclusions

Gentamicin was successfully loaded into the alginate and alginate-chitosan beads. The surface irregularity and size of the beads varied according to the chitosan content. All the GM-loaded alginate-chitosan and alginate beads had obvious antibacterial activity. Alginate-chitosan beads loaded with GM reached about 95% GM release within 3 h, while alginate beads loaded with GM reached about 85% of GM release after 9 h. The amount of GM release was less for crosslinked alginate-GM beads than for uncross-linked beads. The release time and release rate of GM could be manipulated by the chemical structure modulation between alginate, chitosan, gentamicin, and citric acid and the difference in surface roughness and degree of swelling. Controlled gentamicin release through biopolymer conjugation is expected for local disinfection reducing the side effects of systemic antibiotics delivery.

## Data Availability

The data presented in this study are available on request from the corresponding author.
